# How Does Transcranial Magnetic Stimulation Influence Glial Cells in the Central Nervous System?

**DOI:** 10.3389/fncir.2016.00026

**Published:** 2016-04-05

**Authors:** Carlie L. Cullen, Kaylene M. Young

**Affiliations:** Menzies Institute for Medical Research, University of TasmaniaHobart, TAS, Australia

**Keywords:** oligodendrocyte, astrocyte, neural stem cell, microglia, glia, transcranial magnetic stimulation, neural activity, magnetic

## Abstract

Transcranial magnetic stimulation (TMS) is widely used in the clinic, and while it has a direct effect on neuronal excitability, the beneficial effects experienced by patients are likely to include the indirect activation of other cell types. Research conducted over the past two decades has made it increasingly clear that a population of non-neuronal cells, collectively known as glia, respond to and facilitate neuronal signaling. Each glial cell type has the ability to respond to electrical activity directly or indirectly, making them likely cellular effectors of TMS. TMS has been shown to enhance adult neural stem and progenitor cell (NSPC) proliferation, but the effect on cell survival and differentiation is less certain. Furthermore there is limited information regarding the response of astrocytes and microglia to TMS, and a complete paucity of data relating to the response of oligodendrocyte-lineage cells to this treatment. However, due to the critical and yet multifaceted role of glial cells in the central nervous system (CNS), the influence that TMS has on glial cells is certainly an area that warrants careful examination.

## Introduction

Over 30 years ago Barker et al. (1985) showed that it was possible to non-invasively stimulate neurons by generating a localized magnetic field (MF) that penetrated through the scalp, skull and meninges, to induce an electrical current in the brain. Unlike classical electromagnetic fields (EMF), which are usually static in nature, this technique of transcranial magnetic stimulation (TMS) can be administered in different patterns, which appear to exert specific effects on brain activity. For example, low frequency stimulation dampens neural activity, while high frequency stimulation has an excitatory effect (reviewed in Dayan et al., 2013; Parkin et al., 2015). TMS has since been used as an effective tool for understanding neurophysiology, and has also been utilized in the diagnosis and monitoring of neurodegenerative diseases such as motor neuron disease (MND) and Multiple Sclerosis (MS; reviewed by Caramia et al., 2004; Vucic et al., 2013). More recently the clinical application of TMS has been expanding, due to research demonstrating its therapeutic potential for the treatment of migraines (Lipton et al., 2010), tinnitus (Kleinjung et al., 2005), anxiety and major depressive disorders (George et al., 2010; Mantovani et al., 2010), and stroke (Khedr et al., 2010), as well as the management of neurodegenerative disorders such as Parkinson’s disease (PD; Torres et al., 2015), Alzheimer’s disease (AD; Rabey et al., 2013) and MND (Zanette et al., 2008). The therapeutic effect of TMS is largely attributed to its ability to dampen neuronal hyper-excitability, decrease neuro-inflammation, alter blood-brain-barrier (BBB) permeability, and promote neuronal survival.

While TMS is widely utilized within the clinical setting, and the basic principles of TMS have been well described (reviewed by Hallett, 2007), we lack the requisite understanding of how it regulates biological processes. Essentially there is little non-human experimental data demonstrating how TMS works at the cellular and molecular levels. Research conducted in this area initially investigated the effect that TMS exerts on neuronal function, and revealed that it influences neuronal firing patterns *in vivo* and *in vitro* (reviewed by Müller-Dahlhaus and Vlachos, 2013; Tang et al., 2015). The repetitive magnetic stimulation of mouse entorhino-hippocampal slice cultures (70 mm figure eight coil, 10 Hz, 900 pulses) was reported to enhance the NMDA receptor-dependant recruitment of AMPA receptors to the post-synaptic density to increase glutamatergic synaptic strength (Vlachos et al., 2012). These data may help explain why sub-threshold low-frequency repetitive TMS (rTMS; 25 mm figure eight coil, 1 Hz for 4 min), which would be expected to have the opposite effect, can actually prevent the development of seizures in a rat kindling model of epilepsy (Shojaei et al., 2014). However it is likely that a number of mechanisms are involved, as low-intensity rTMS has also been shown to correct a genetically-programmed aberrant axon guidance phenotype in mice (Rodger et al., 2012; Makowiecki et al., 2014), suggesting that rTMS may influence cytosolic calcium regulation in multiple ways.

Research conducted over the past two decades has made it increasingly clear that a group of non-neuronal cells, collectively known as glia, respond to and facilitate neuronal signaling. Glia make up the majority of cells in the adult brain, far exceeding neurons in number and diversity. Within the adult central nervous system (CNS), they can be divided into five major cell types: adult neural stem cells, which generate new neurons that are required for learning and memory (reviewed by O’Rourke et al., 2014); astrocytes, which perform a diverse range of functions, including neurotransmitter uptake and the buffering of extracellular potassium ion concentration (reviewed by Clarke and Barres, 2013); oligodendrocytes, which support axons through myelin production and the provision of trophic support (reviewed by Nave, 2010); oligodendrocyte progenitor cells (OPCs), which proliferate and generate new oligodendrocytes (reviewed by Richardson et al., 2011); and microglia, which are the resident immune cells (Tambuyzer et al., 2009). Each glial cell type has the ability to respond to electrical activity directly or indirectly, making them likely cellular effectors of TMS—a possibility that will be explored in this review.

## Neural Stem Cells Respond to TMS

Neural stem cells are located within the dentate gyrus of the hippocampus and the subventricular zone of the mature brain in both humans and rodents (reviewed in Ming and Song, 2011). They can be identified by their expression of glial fibrillary acidic protein (GFAP), a protein generally associated with astrocytes, as well as nestin, and sox2 (Lugert et al., 2010), and are relatively quiescent, dividing infrequently to produce intermediate progenitor cells, which are rapidly dividing, and in turn generate neuroblasts (Silva-Vargas and Doetsch, 2014). A fraction of adult-born neurons survive, mature and synaptically integrate into the neural network as functionally mature neurons (Fuentealba et al., 2012; Silva-Vargas et al., 2013). Proliferation in the neural stem cell niche is known to be regulated by neuronal activity (reviewed Kempermann, 2015), and a number of studies indicate that rTMS can drive neural stem cell proliferation and neurogenesis. For example, the application of rTMS to adult mice, every day for 2 weeks (100 mm diameter coil, 150 pulses per day), at either 1 Hz or 30 Hz, increased the number of neural stem/progenitor cells present in the subventricular zone (SVZ), suggesting that rTMS induced proliferation and an expansion of the population (Abbasnia et al., 2015). A similar increase in neural stem and progenitor cell (NSPC) proliferation was detected when TMS was directed towards the hippocampus *in vivo*, irrespective of whether it was the deep-brain magnetic stimulation of mice (20 min successive trains of 500 ms pulses administered daily; Zhang et al., 2014) or the chronic rTMS treatment of rats (70 mm figure 8 coil, 25 Hz, 14000 pulses for 14 consecutive days; Ueyama et al., 2011).

The ongoing addition of highly plastic immature neurons to the hippocampus is crucial for learning and memory (Denny et al., 2012; Garthe and Kempermann, 2013)— making this adult neural stem cell niche an interesting target for the treatment of dementia (Ager et al., 2015). However, new hippocampal neurons are also thought to influence mood, making the ability of TMS to stimulate neurogenesis also particularly relevant to the positive effect that rTMS has when used to treat patients with depression. One of the current working models of this disease links a decrease in hippocampal volume, stem cell activity and neurogenesis with the establishment of depression, and its reversal with the success of anti-depressant therapies (reviewed by Chaudhury et al., 2015; Miller and Hen, 2015). The antidepressant drug Quetiapine and deep brain TMS (7 cm diameter magnet pair, 60 Hz, 0.7T, 2 h twice daily) are both able to increase neural stem cell proliferation in the dentate gyrus of chronically stressed rats. Alone neither treatment maximally stimulates NSPC proliferation, as their joint application can further increase the number of 5-bromo-2′-deoxyuridine (BrdU^+^) cells present in the dentate gyrus (Chen et al., 2015). However the magnitude of this increase is small, suggesting that limited therapeutic benefit would be obtained by combining these treatments.

Most of the BrdU labeling studies that examine the effect of TMS on neural stem cell activity and neurogenesis, do not distinguish between the effect of TMS on neural stem cell proliferation vs. new cell survival. Therefore more detailed studies are needed to elucidate the mechanisms underlying the influence that TMS exerts on this cell type. However it is clear that numerous stimulation methods and patterns of TMS can enhance adult neurogenesis from both stem cell niches. Of particular interest for NSPC regulation, the application of TMS has been linked to changes in the expression of microRNA (miR)-25. Mir-25 has been previously shown to promote neural stem cell proliferation (Brett et al., 2011) by blocking the expression of a cyclin-dependent-kinase inhibitor, p57 (Guo et al., 2014). However the repetitive magnetic stimulation of cultured P3 rat hippocampal neural stem cells, which also enhances their proliferation (≥800 pulses from a 90 mm figure eight coil), is reported to reduce miR-25, but increase miR-93 and miR-106b (Liu et al., 2015), which instead target the cell cycle regulators integrin-β8 and p21 (Kim et al., 2009; Fang et al., 2011). While the exact regulators may vary between experimental conditions, it appears likely that TMS influences NSPC proliferation by altering gene expression.

The application of TMS for the treatment of dementia and mood disorders seems logical, given the natural function of the new neurons being added to the hippocampus, however TMS may also allow the manipulation of NSPC behavior to enact nervous system repair more broadly. Deep-brain magnetic stimulation has been shown to increase the number and length of dendrites on newly generated dentate granule neurons (Zhang et al., 2014), suggesting that it can influence the maturation of new neurons. Furthermore TMS maybe suitable to promote remyelination. New oligodendrocytes are produced from neural stem cells in the mouse subventricular zone (Young et al., 2010), and they are produced in larger numbers in response to cuprizone-mediated demyelination of the corpus callosum (CC; Xing et al., 2014). In a similar focal model of CC demyelination, mice exposed to oscillating EMF (7 cm diameter magnet pair, 60 Hz, 0.7T, 2 h twice daily from injury) had a larger number of BrdU^+^ newborn cells present in the CC and more nestin^+^ cells in the subventricular zone, relative to control mice (Sherafat et al., 2012). These changes in NSPC activity were accompanied by a less severe demyelination, prompting the authors to suggest that this intervention had promoted neural stem cell-mediated repair. TMS may be similarly beneficial following stroke, as rats receiving supra-threshold rTMS, to target the ipsilateral motor cortex (6 cm, 3.5T coil, 10 Hz for 300 pulses, daily for 7 days) following middle cerebral artery occlusion, showed enhanced NSPC proliferation in the underlying subventricular zone (Guo et al., 2014). However the longer-term benefits of this treatment have not been examined. More research is clearly needed to define the outcomes of TMS treatment in these different disease models and define the signaling mechanisms that are downstream of TMS, including those regulating NSPC proliferation. However there are a number of likely mechanisms, which we have outlined below.

### TMS-Mediated Neurotransmitter Release as a Driver of Adult Neurogenesis

Two key signaling molecules regulating neural stem cell proliferation within the hippocampus are GABA and glutamate. GABA is released from parvalbumin-positive interneurons and can “spill over” and act on GABAγ2 receptors on neural stem cells to maintain their quiescence (Song et al., 2012). Therefore, a reduction in parvalbumin-positive interneuron activity would be expected to promote neural stem cell proliferation. Furthermore, using an alternative method of stimulation, it has been shown that the induction of hippocampal long-term potentiation (LTP) enhances the production of new neurons from hippocampal NSPCs in rats (Cho et al., 2013), and rTMS would be expected to induce LTP in some hippocampal neurons and produce a similar response. In mice, the activity-dependent increase in neurogenesis can be blocked using mGluR5 antagonists, and mimicked using a blood-brain-barrier permeable mGluR5 agonist (Nochi et al., 2012). Therefore it would interesting to determine the importance of mGluR5 in mediating TMS-stimulated neurogenesis.

Alternatively, serotonin release from neurons in the raphe nucleus, can act on 5HT-2C and 5HT-2A receptors on neural stem cells situated in the subventricular zone, depolarizing the cells, and ultimately promoting their proliferation (Tong et al., 2014). Similarly the release of serotonin in the hippocampus can promote hippocampal NSPC proliferation (reviewed Alenina and Klempin, 2015). Therefore it is possible that TMS promotes neurotransmitter release from serotonergic neurons, either directly or indirectly, and thereby increases NSPC proliferation. Activation of the g-protein coupled serotonergic receptors would also be expected to trigger an increase in cytoplasmic calcium, which could also modulate gene transcription in the NSPCs, to facilitate neurogenesis. *In vitro* neural stem cells connect via connexin-43 gap junctions, which allow them to propagate electrical signals between neighboring cells, activating voltage-gated calcium channels and triggering calcium oscillations (Malmersjö et al., 2013). The shRNA-mediated knockdown of connexin-43 from neural stem cells during mouse development significantly reduced stem cell proliferation (Malmersjö et al., 2013). Therefore, if TMS increases neurotransmitter release from neurons, which acts on neural stem cells, this altered activity may be experienced across the neural stem cell network, feasibly resulting in a calcium signal that would be sufficient to alter transcription.

### TMS-Induced Neurotrophin/Growth Factor Release as a Driver of Adult Neurogenesis

rTMS increases BDNF and VEGF expression levels in the hippocampus and cortex of rat brains (Müller et al., 2000; Zhang et al., 2015), and the depolarization of cultured hippocampal neurons by repetitive magnetic stimulation induces the release of BDNF from their dendrites (Waterhouse et al., 2012). Similarly cultured neuroblastoma cells have been shown to release BDNF, NT3, GDNF and PDGF-A in response to repetitive magnetic stimulation (Lee et al., 2015). BDNF, VEGF and NT-3 are known regulators of adult neurogenesis. *In vivo* the dendritic release of BDNF dampens neural stem/progenitor cell proliferation, but enhances the survival of the new neurons (Waterhouse et al., 2012). Whereas, VEGF has been reported to bind the VEGF receptor 3 to facilitate NSPC division and neurogenesis in the hippocampus of mice and humans (Han et al., 2015), but has also been shown to promote new cell survival without altering proliferation in the rat brain (Schänzer et al., 2004). Unlike BDNF and VEGF which are both pro-neurogenic signals, NT-3 is secreted from endothelial cells in the subventricular zone and is a neural stem cell quiescence signal (Delgado et al., 2014; Silva-Vargas and Doetsch, 2014), making it unlikely that NT-3 is a dominant signal in the neural stem cell niche following TMS.

## Astrocytes Respond to Electrical Activity and Facilitate Neuronal Signalling

Astrocytes are important regulatory cells within the CNS and are likely to be critical mediators of TMS-induced brain changes. Astrocytes provide metabolic support for neurons, shuttling lactate through monocarboxylate transporters (Bélanger et al., 2011; Choi et al., 2012), they actively communicate with neurons in a reciprocal manner at the so called “tripartite synapse” (Santello et al., 2012), and they play a vital role in synapse formation and function (reviewed by Clarke and Barres, 2013). In the normal healthy brain, GFAP is a marker of fibrous astrocytes (Young et al., 2010). Following a CNS injury the expression of this protein can be up-regulated, even by protoplasmic astrocytes (Nolte et al., 2001). However the change in GFAP expression can be highly variable (Zamanian et al., 2012), making it an imperfect marker of astrogliosis. Despite this, GFAP has been used to examine astrogliosis in the TMS field, as a way of assessing treatment safety.

The acute magnetic stimulation of cultured astrocytes induces a transient increase in GFAP levels that lasts for 3 days (10 Hz for 10 s; Chan et al., 1999). rTMS has also been shown to induce a transient increase in GFAP expression *in vivo*, when treatment was applied following ischemic injury (continuous 50 Hz, 0.5 mT exposure for 7 days; Rauš et al., 2013) or to a demyelinated lesion (1 Hz for 5 min per day for 14 days; Fang et al., 2010). By contrast, rTMS was found to attenuate astrocyte activation at the site of spinal cord (SC) injury in rats (25 Hz, 3 s on/off for 20 min, 5 days a week for 8 weeks; Kim et al., 2013). Furthermore rTMS (six trains of 300 pulses daily for 18 days, 20 Hz) did not alter GFAP expression in the dentate gyrus of rats experiencing chronic stress (Czéh et al., 2002), or alter the number of GFAP-positive cells in the motor cortex or hippocampus of normal, healthy rats (1000 pulses per day for 5 days at 1 Hz; Liebetanz et al., 2003). Astrogliosis is often accompanied by a morphological change in the astrocytes, and this was observed following direct current stimulation of cultured astrocytes (Pelletier et al., 2014), but was not observed following magnetic stimulation (Chan et al., 1999). These data indicate that TMS can effect astrocytes, however this effect is highly context-dependent, and is therefore likely to be a secondary effect and reliant on TMS influencing another cell-type in the environment.

Astrocytic function is influenced by changes in neuronal activity, they are key cellular components of synapses, and they release factors that also influence synapse number. Given what we know about astrocytic function, the observation that rTMS alters neuronal synapse number in the CNS (Vlachos et al., 2012), strongly implicates astrocytes as cellular effectors of TMS. Neurotransmitter release from neurons is sensed by astrocytes, which respond with an increase in intracellular calcium, and the subsequent release of gliotransmitters (see Paixão and Klein, 2010 for review). The addition of astrocytes or astrocyte-conditioned media to cultured neurons is known to increase the number of functional excitatory synapses formed in the culture, while the removal of astrocytes or their secreted signals results in a rapid reduction in functional synapse number (Ullian et al., 2001). Some of the secreted factors responsible have been identified, and include members of the thrombospondin (TSP) family, namely TSP1, which *in vivo* is thought to be secreted in response to release of the neurotransmitter ATP (Tran and Neary, 2006), TSP2 and TSP4. TSPs bind to the α_2_δ-1 subunit of voltage-gated calcium channels, causing a conformational change in this subunit, which triggers an uncharacterized signaling cascade, leading to the recruitment of adhesion and scaffolding molecules to the synaptic site (Eroglu et al., 2009). Hevin [also known as secreted protein acidic and rich in cysteine (SPARC)-Like protein 1] was also shown to promote excitatory synapse formation, while its homolog— SPARC, is antagonistic to this process (Kucukdereli et al., 2011). Astrocytes also control synapse number by engulfing and eliminating synapses—a process that is strongly regulated by neuronal activity (Chung et al., 2013). Therefore it would be interesting to determine whether the release of these factors is increased following TMS, or whether there is any change in astrocyte-mediated phagocytosis.

In addition to mediating synapse formation, astrocytes are likely to affect changes in spine shape in response to TMS. The repetitive magnetic stimulation of hippocampal slice cultures has been reported to induce the clustering of post-synaptic AMPA receptors, as well as the enlargement of the post-synaptic terminals (Vlachos et al., 2012; Lenz et al., 2015). This is likely mediated by Ephrin A3 expressed in astrocytic processes, and its receptor EPHA4 which is highly expressed by dendritic spines (Filosa et al., 2009). Inhibition of ephrin A3-EPHA4 interaction has been shown to distort spine shape and organization, suggesting a role for astrocytes in regulating neuronal morphology (Murai et al., 2003). Furthermore astrocytes regulate synaptic maturation and excitability via the secretion of glypicans, which increase the insertion of Glu1A-containing AMPA receptors into the post-synaptic membrane, leading to greater excitability of the post-synaptic neuron (Allen et al., 2012). Additionally, the release of glutamine from astrocytes is critical for the sustained release of glutamate from neurons (Tani et al., 2014). The therapeutic effect of TMS in counteracting depression has been largely attributed to the potentiation of glutamatergic transmission (Croarkin et al., 2016), and these data provide a strong case for the involvement of astrocytes in mediating this effect of TMS on synaptic structure and efficacy.

Astrocytes further modulate synaptic function through their uptake of the potassium released by neurons during action potentials, and through their uptake of glutamate and GABA from the synaptic cleft (reviewed by Anderson and Swanson, 2000). Glutamate uptake into astrocytes occurs via the VGLUT1 transporter, which is regulated in a dose-dependent manner in the frontal, motor, somatosensory and visual cortices of rats, by the application of rTMS (70 mm figure eight coil, continuous or intermittent TBS, 600–2400 pulses; Volz et al., 2013). While this study did not look at the cell-type specific levels of VGLUT1, this receptor is highly expressed by astrocytes, and therefore a change in expression by these cells would be necessary to observe this overall effect. Therefore, in response to TMS, it is likely that the increased neuronal firing is detected by astrocytes, and that they modulate VGLUT1 expression to ensure their sustained uptake of synaptic glutamate.

## Microglia Modulate Synaptic Plasticity

Microglia are the resident immune cells of the CNS, and they play a multifaceted role in modulating synaptic plasticity. The effect of TMS on microglia *in vivo* has been largely unexplored. In normal healthy rats, the application of high intensity, low frequency rTMS does not affect microglial number in the motor cortex or hippocampus (Liebetanz et al., 2003). However the application of very low intensity, but high frequency rTMS following an ischemic injury, or the induction of demyelination, appears to activate microglia, leading to increased Iba1 expression (Fang et al., 2010; Rauš et al., 2013). In contrast, high intensity, high frequency rTMS applied to the injured SC, reportedly attenuates microglial activation (Kim et al., 2013). While it is not possible to gauge the effect of TMS on microglia from such a small number of studies, TMS would be expected to affect microglial behavior. Microglia are known to preferentially phagocytose weak or inactive pre-synaptic terminals, guided by the activity-dependent expression of complement (Schafer et al., 2012; reviewed by Morris et al., 2013). Furthermore, microglia secrete BDNF and the cytokine interleukin 1β, which have been shown to facilitate LTP (Rogers et al., 2011; Ferrini and De Koninck, 2013). As microglia influence neuronal activity, and respond to signals that are altered by neuronal activity, their role as cellular mediators of TMS warrants further investigation.

## Cells of the Oligodendrocyte-Lineage Respond to Electrical Stimulation

### How do Mature Oligodendrocytes Respond to TMS?

Oligodendrocytes are the cells that myelinate axons in the CNS. As an oligodendrocyte matures, it extends its myelin membrane to wrap around multiple axons. Following this process of ensheathment, the membrane compacts to form the functional myelin internode (Snaidero et al., 2014). Each internode increases the membrane resistance and decreases the capacitance of the axon segment it surrounds, and they are vital for the saltatory conduction of action potentials along the axon. While the effect of TMS on oligodendrocytes has not been investigated, they are known to express a range of neurotransmitter receptors and ion channels (reviewed by Káradóttir and Attwell, 2007). Furthermore, recent studies suggests that they can sense and respond to neuronal activity, and adjust the properties of their myelin sheath to modulate conduction velocity (Yamazaki et al., 2007, 2014). This dynamic regulation of conduction velocity by oligodendrocytes is dependent on the magnitude of depolarization (Yamazaki et al., 2014). The large surface area of oligodendrocytes may allow TMS to directly induce a current in these cells, as the application of TMS to neurons can increase intracellular calcium to levels equivalent to that seen post action potential firing (Grehl et al., 2015). However it seems more likely that any TMS-mediated effect on oligodendrocytes would be indirect and act to trigger the release of calcium from intracellular stores. While white matter abnormalities are closely associated with a number of psychiatric disorders (reviewed by Fields, 2008), the benefit of oligodendrocyte depolarization is physiologically unclear, making it difficult to speculate how this phenomenon might relate to the therapeutic benefits of TMS in the treatment of neurological disorders.

### Can TMS Influence the Behavior or OPCs and Promote Oligodendrogenesis?

TMS may also effect myelination by the indirect stimulation of OPCs - the population of cells that give rise to mature oligodendrocytes throughout life (Richardson et al., 2011). OPCs are unique glial cells, in that they receive direct synaptic input from neurons (Bergles et al., 2000; Lin and Bergles, 2004; Kukley et al., 2007; Ziskin et al., 2007). In recent years it has been shown that theta burst firing of the pre-synaptic neuron can trigger the insertion of calcium-permeable AMPA receptors (glutamate receptors) at the OPC post-synaptic density, in a process termed glial LTP (Zonouzi et al., 2011). Additionally, blocking neuronal action potentials *in vivo* decreases OPC proliferation (Barres et al., 1993) and oligodendrocyte production (Demerens et al., 1996), while the repeated stimulation of neurons in the motor cortex (using a tripolar electrode) promotes the activity-dependent proliferation of OPCs in the corresponding corticospinal tract (Li et al., 2010). Increased axonal myelination also occurs *in vitro* following the frequency-dependant electrical stimulation of neurons (Malone et al., 2013). Similarly the direct current stimulation of oligodendrocyte and neuron co-cultures enhanced the survival and myelinating capacity of the oligodendrocytes (Gary et al., 2012). More recently, *in vivo* optogenetic stimulation (20 Hz) of projection neurons in the pre-motor cortex demonstrated that increased neuronal firing was accompanied by increased OPC proliferation, oligodendrocyte generation, and the addition of thicker myelin to the axonal projections extending from the premotor cortex to the CC (Gibson et al., 2014). Given the ability of TMS to increase neuronal firing, these data would strongly suggest that TMS would enhance oligodendrogenesis in this way. However the influence of TMS on oligodendrogenesis may be two fold. There is mounting evidence that TMS increases BDNF levels in the CNS, and BDNF is known to bind to tropomyosin receptor kinase B on immature oligodendrocytes to regulate axonal ensheathment (Xiao et al., 2010; Wong et al., 2013). Therefore it is likely that TMS enhances oligodendrogenesis in adulthood, but that each of these effects are secondary to the influence that TMS exerts on neuronal activity.

## Conclusions and Future Perspectives

The use of TMS and other activity-based therapies is increasing, and despite the important role played by glial cells in responding to activity and regulating activity in the CNS, they have been largely overlooked in this field, with only a small number of studies examining the role of TMS on glia *in vivo* (see Table 1). It is likely that many of the beneficial effects of TMS are the result of the secondary activation of glial cells. To fully understand the therapeutic benefits that can be obtained through the application of TMS, it will be critical to understand which stimulation patterns most influence glial cell populations, perhaps even opening up previously unconsidered therapeutic options for the use of TMS to manipulate glial cell function.

**Table 1:** **Summary of current literature reporting the effect of TMS on glia *in vivo***.

Reference	Model	Method of stimulation	Field intensity	Stimulation pattern	Stimulation duration	Post stim assessment	Region assessed	Main effect of TMS
Czéh et al. (2002)	Psychosocial stress in rats	rTMS	4T (130% motor threshold)	20 Hz for 300 pulses (2.5 s)	18 days	24 h	Dentate Gyrus	Attenuates stress- induced ↓ in NSPC proliferation. Potentiates ↓ cell survival.
Liebetanz et al. (2003)	Normal, healthy rats	rTMS Round coil	7T	1 Hz, for 1000 pulses (reversed current flow after 500 pulses)	5 days	48 h	Motor cortex and Hippocampus	No change in astrocyte or microglia density
Arias-Carrión et al. (2004)	Nigrostriatal lesion and chromaffin cell transplant in rats	Oscillatory magnetic field via two 7 cm Hemholtz coils positioned dorsal and ventral to the head	0.7 mT	60 Hz, 2 h morning and afternoon	7 or 60 days	Same day	SVZ	↑ NSPC proliferation.
Dwork et al. (2009)	Magnetic seizure therapy in *Macaca mulatta*	rTMS Round coil	2.5 × seizure threshold	50 Hz, 8 s trains	4 days/week for 6 weeks	3 days	Hippocampus and PFC	No change in glial density
Fang et al. (2010)	Ethidium bromide injury in rats	rTMS Round coil	0T, 0.76T, 1.52T, 1.9T	1 Hz, 5 min/day	14 days	14 days	Spinal cord	↓ lesion size ↑ GFAP
Ueyama et al. (2011)	Normal, healthy rats	rTMS Figure-of-eight coil	70% maximum power	25 Hz, 4 × 10 s trains daily	14 days	1 day	Dentate Gyrus	↑ NSPC proliferation ↑ neurogenesis
Sherafat et al. (2012)	Lysolecithin-induced demyelination of the rat CC	Oscillatory magnetic field. 2 × 7 cm Hemholtz coils positioned dorsal and ventral to the head	0.7 mT	60 Hz, 2 h morning and night	7,14 or 28 days	Same day	CC and SVZ	↑ NSPC proliferation ↑ MBP
Rauš et al. (2013)	Ischemic injury in gerbils	Alternating geomagnetic field	0.2–2 mT (0.5 mT at cage center)	50 Hz	7 days	7 days	Hippocampus (injury site)	↑ GFAP + astrocytes ↑ Iba1 + microglia
Kim et al. (2013)	Spinal cord injury in rats	rTMS Round coil	200 mT	25 Hz, 3 s on/off for 20 min	5 days/week for 8 weeks	Same day	Spinal cord	Attenuates astrocyte and microglial activation
Zhang et al. (2014)	Normal, healthy mice	Deep brain magnetic stimulation via two coils placed either side of the cage	10 mT peak	Varying pulsed magnetic fields	4 or 7 days	1 day	Dentate Gyrus	↑ NSPC proliferation ↑ Neurogenesis
Abbasnia et al. (2015)	Normal, healthy mice	rTMS Round coil	not specified	1 Hz or 30 Hz for 150 pulses	7 or 14 days	1 day	SVZ	Larger neurospheres produced from SVZ stimulated for 14 days
Chen et al. (2015)	Chronic unpredictable stress in rats	rTMS Round coil	1.26T	15 Hz, 15 s trains, 900 pulses daily	7 days	1 day	Dentate Gyrus	↑ BDNF expression ↑ NSPC proliferation

*mT, millitesla; T, Tesla; MF, magnetic field; rTMS, repetitive transcranial magnetic stimulation; Hz, hertz; SVZ, sub ventricular zone; CC, corpus callosum; SC, spinal cord; BrdU, 5-bromo-2’-deoxyuridine; NSPC, neural stem and/or progenitor cell; PFC, prefrontal cortex*.

## Author Contributions

CLC and KMY carried out the research and wrote the article.

## Conflict of Interest Statement

The authors declare that the research was conducted in the absence of any commercial or financial relationships that could be construed as a potential conflict of interest.
